# A ‘combined framework’ approach to developing a patient decision aid: the PANDAs model

**DOI:** 10.1186/s12913-014-0503-7

**Published:** 2014-10-24

**Authors:** Chirk Jenn Ng, Nigel Mathers, Alastair Bradley, Brigitte Colwell

**Affiliations:** Department of Primary Care Medicine, Faculty of Medicine, University of Malaya, Kuala Lumpur, 50603 Malaysia; Academic Unit of Primary Medical Care, School of Medicine & Biomedical Sciences, Sam Fox House, Northern General Hospital, Herries Road, Sheffield, UK

**Keywords:** Patient decision aids, Decision making, Diabetes mellitus, Insulin, Complex intervention

## Abstract

**Background:**

There is a lack of practical research frameworks to guide the development of patient decision aids [PtDAs]. This paper described how a PtDA was developed using the International Patient Decision Aids (IPDAS) guideline and UK Medical Research Council (UKMRC) frameworks to support patients when making treatment decisions in type 2 diabetes mellitus.

**Methods:**

This study used mixed methods to develop a PtDA for use in a UK general practice setting. A 10-member expert panel was convened to guide development and patients and clinicians were also interviewed individually using semi-structured interview guides to identify their decisional needs. Current literature was reviewed systematically to determine the best available evidence. The Ottawa Decision Support Framework was used to guide the presentation of the information and value clarification exercise. An iterative draft-review-revise process by the research team and review panel was conducted until the PtDA reached content and format ‘saturation’. The PtDA was then pilot-tested by users in actual consultations to assess its acceptability and feasibility. The IPDAS and UKMRC frameworks were used throughout to inform the development process.

**Results:**

The PANDAs PtDA was developed systematically and iteratively. Patients and clinicians highlighted the needs for information, decisional, emotional and social support, which were incorporated into the PtDA. The literature review identified gaps in high quality evidence and variations in patient outcome reporting. The PtDA comprised five components: background of the treatment options; pros and cons of each treatment option; value clarification exercise; support needs; and readiness to decide.

**Conclusions:**

This study has demonstrated the feasibility of combining the IPDAS and the UKMRC frameworks for the development and evaluation of a PtDA. Future studies should test this model for developing PtDAs across different decisions and healthcare contexts.

**Electronic supplementary material:**

The online version of this article (doi:10.1186/s12913-014-0503-7) contains supplementary material, which is available to authorized users.

## Background

Shared decision making is an important component of patient-centred care [1]. It involves the exchange of information and negotiation between the patients and healthcare professionals to reach a consensus on a particular decision [2]. The decision making process is complex and different decision support interventions have been developed to facilitate this process [3].

Patient decision aids (PtDAs) are ‘interventions designed to help people make specific and deliberative choices among options (including the status quo) by providing information on the options and outcomes relevant to a person’s health status and implicit methods to clarify values’ [4]. They are different from patient health education materials which provide general health information about specific medical conditions including diagnosis, investigation and treatment. There are currently more than 500 PtDAs that have been developed worldwide, mainly in North America and Europe. [Decision Aids Library Inventory http://decisionaid.ohri.ca].

The effectiveness and utility of a PtDA is dependent on its quality. The International Patient Decision Aid Standards (IPDAS) collaboration has identified appropriate quality indicators using a Delphi consensus method and this provides guidance on the assessment of the content, format and evaluation of the PtDA (Table 1) [5]. However, few PtDAs developers describe the development process per se and, of those which do, there is a wide variation in how PtDAs are actually developed. In addition, PtDAs are often developed without users’ involvement [6].

**Table 1:** **Development of the PANDAs insulin PDA using the IPDAS collaboration framework** [6]

**IPDAS criteria**	**Domains**	**PANDAs insulin PDA**
**1. Providing information about options**	Development (content)	• The information included in the PDA was based on two criteria:
		○ What do patients want to know before making a decision
		○ What do patients need to know before making a decision?
		• The findings from the needs assessment of the patients informed what and how much they wanted to know before making a decision
**2. Presenting probabilities**	Development (content)	• The “risk communication” section of the PDA was based on the decision making theories
		○ Use of event rates specified by the population in period (e.g. number of people affected out of 100 people over 5 years)
		○ Comparison of outcomes probabilities using the same denominator, period, scale (e.g. out of 100 people over 5 years)
		○ Description of the uncertainty around probabilities (‘platinum’, ‘gold’, ‘silver’ and ‘bronze’)
		• Visual diagrams (“smiley” faces) were used in conjunction with other methods to illustrate the probabilities (words, numbers, diagram)
		• ‘Smiley’ sticker was used to present individualised risk to patients based on their HbA1c
**3. Clarifying and expressing values**	Development (content)	• Values may be attributed to
		○ Each treatment option (e.g. values attributed to “make no change”, “more adherent to existing treatment” and “ starting insulin”)
		○ Specific features of the treatment option i.e. the value of the procedure/process (e.g. the values associated with insulin injection)
		○ Value of outcomes (e.g. the values associated with weight gain due to insulin treatment)
		○ Value of probabilities (e.g. the values associated with the probabilities of gaining 6-8 lbs in weight over a year with insulin treatment).
		• The PANDAs insulin PDA helped patients to clarify their own values using an explicit approach
		• Patients worked through a personal worksheet in the PDA to determine how important each feature and outcome of the treatment options were to them
**4. Guiding/coaching in deliberation and communication**	Development (content/format)	• IPDAS quality criteria recommend that PDAs should:
		○ Provide steps to make a decision
		○ Suggest ways to talk about the decision with a health professional
		○ Include tools (e.g. workshop question list) to discuss options with others.
		• The PANDAs PDA
		○ Provides a five-step systematic approach to decision making (Table 3)
		○ Encourages the patient to discuss uncertainties or queries with the healthcare professionals (prompts)
		○ Encourages the patient to write down their questions for the healthcare professionals
**5. Disclosing conflicts of interest**	Development (process)	
**6. Balancing the presentation of options**	Development (content/format)	• PANDAs insulin PDA provided balanced information by
		○ Making comparison of the positive and negative features of each option
		○ Both features were presented with equal detail and in the same format (font, order, display of statistic)
		• The balance of the PDA was assessed during the acceptability study by asking the patients and the healthcare professionals how balanced and fair they found the information presented
**7. Using plain language (readability)**	Development (format)	• The PANDAs insulin PDA used “Simply Put” plain language guideline produced by the Centre for Communicable Disease and Prevention (1999).
		• The “readability” was assessed using the Readability Calculations, version 7.0 software (which includes the SMOG and FRY readability tests)
		• Patients and patient education experts also reviewed the PDA
**8. Basing information on up to date scientific evidence**	Development (content/process)	• Where possible, clinical evidence based on systematic reviews and national or local clinical practice guidelines were used.
**9. Establishing effectiveness**	Evaluation	

**Figure 1:**
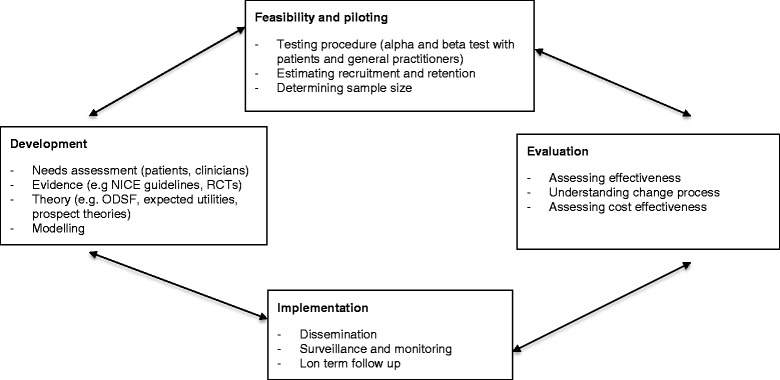
**Modification of the UKMRC framework for the development and evaluation of complex interventions [** 7 **].**

Although the IPDAS guideline provides a framework for the development of a PtDA, it does not provide guidance on which research methods to use; neither does it explicitly link the development of a PtDA to its evaluation and implementation. It is, therefore, necessary to combine these guidelines with other frameworks such as that produced by the UK Medical Research Council (UKMRC) framework which can link the development of the PtDA to its evaluation and implementation (Figure 1) [7,8]. It is designed to guide the development and evaluation of complex interventions, such as PTDAs, and it stresses the important role of evidence, theories and modelling in the process of developing a complex intervention. Specific research methods are recommended to operationalise each step and it was for this reason that UKMRC Framework was used to complement the IPDAS guideline to develop and evaluate the PDA described in this study. In addition, the UKMRC framework highlights the importance of having a range of options before deciding which intervention to use and that it should be based on existing best available evidence. Before developing a decision support tool, it is important to reflect whether PtDA is the best tool to use to support decision making.

This paper described how the Patient and insulin Decision Aids (PANDAs) PtDA was developed for people with Type 2 Diabetes Mellitus [T2DM] based on the IPDAS criteria and UKMRC framework. The PANDAs study was conducted from 2007–2010 to develop the PtDA and to evaluate its effectiveness in the UK general practice [9].

## Methods

We used a systematic and iterative approach to develop the PtDA by incorporating the IPDAS and UKMRC framework (Table 2). The UKMRC framework informed the methods used for the development of the PtDA, which is not stated in the IPDAS guideline. This section described in detail how the methods proposed by the UKMRC framework were used to operationalise each step: expert consensus (e.g. drafting of the PtDA), literature review (e.g. collating clinical evidence), individual interviews (e.g. needs assessment) and questionnaire surveys (e.g. acceptability and feasibility) (Table 2). This part of the PANDAs study was conducted in the general practice setting, Sheffield, England in 2007–2008. We obtained ethics approval from the National Health Service Research Ethics Committee (REC reference: 07/Q2308/53).

**Table 2:** **The development process of the PANDAs insulin PDA**

**Steps**	**Framework**	**Objectives**	**Methods**	**Outcome**
**1. Convene an expert panel**	UKMRC/IPDAS	• To guide the development of the PDA, including determining the clinical focus, needs assessment, research methods, content and format of the PDA, as well as evaluation and implementation	• Expert consensus (face-to-face meetings)	• Ten stakeholders were selected, including general practitioners, diabetologist, diabetes educator, expert patients, representative from Diabetes UK, patient decision support experts, statistician
				• Four meetings were conducted during the one-year period
**2. Assess users’ needs**	UKMRC/ IPDAS	• To assess the needs of patients with type 2 diabetes who are making treatment decisions	• Individual patient and clinician interviews	• Nine patients at the point of decision making and 14 general practitioners, nurses and dieticians involved in diabetes care were interviewed
		• To assess the needs of clinicians who are supporting patients’ decision making		• The users identify decisional, emotional, information and social support needs
		• To determine the preferred decision support tool and its mode of delivery		• A paper-based decision support tool is preferred
**3. Review the literature**	UKMRC/ IPDAS	• To identify the range of effective decision support tools (general)	• Literature review	• PDA was selected as the decision support tool with the most evidence [10]
		• Identify existing decision support tool for diabetes treatment (specific)		• Decision support tools were identified [Decision Aid Library Inventory http://decisionaid.ohri.ca]
**4. Identify the theoretical framework**	UKMRC	• To review the existing decision support theories	• Literature review	• Ottawa decision support framework was selected as it was the most used and implemented [11]
**5. Collate the clinical evidence for treatment options**	IPDAS	• To search, select and synthesise the evidence of the pros and cons of the treatment options	• Literature review, focusing on systematic reviews and local/national clinical practice guidelines	• There was a lack of systematic reviews on the efficacy and safety of insulin vs oral oral hypoglycaemic agents. Evidence was synthesized from cohort studies [12]
**6. Drafting of the PDA (Alpha testing I)**	IPDAS/UKMRC	• The design the PDA (content and format) team and PDA design expert drafted the PDA iteratively	• Draft-review-revise iterative process by the research team and PDA design experts	• The preliminary draft of the PDA was developed based on the IPDAS criteria and went through 13 iterations between the researchers and PDA design experts
**7. Review by the expert panel (Alpha testing II)**	IPDAS/UKMRC	• To review the PDA by the stakeholders (not part of research team)	• Expert panel consensus (meetings and emails)	• The research team and the PDA design experts discussed the feedback and agreed on the final draft for beta testing
**8. Develop the PDA training module for clinicians**		• To develop a training module, including a guidebook and workshop, to guide clinicians on how to use the PDA with the patients	• Expert consensus involving research team, decision support experts, diabetes educator and medical education expert	• A PDA guidebook for clinicians
				• A 1-2 hour workshop involving short lectures, demonstration and feedback
**9. Assess the readability**	IPDAS	• To assess the readability of the PDA	• Readability Calculations v7.0 software	• The readability was at grade 8 (or English year 9) using SMOG and Fry
**10. Review by patients and clinicians (Beta testing)**	IPDAS	• To assess the acceptability and feasibility of the PDA in real consultations	• Patient and clinician questionnaire survey	• Nine patients and 14 clinicians found the PDA acceptable and feasible
			• Individual interviews with patients and clinicians	
**11. Finalise the PDA**	IPDAS	• To finalise the content, design and quantity to be printed	• Research team and PDA design expert consensus	• A 16-paged paper PDA was developed
		• To declare conflict of interest, next update		

The members of the expert panel were selected based on their experience as patients or involvement in the care for people with type 2 diabetes in general practice (Table 2). The expert panel was responsible for reviewing the first and the subsequently revised drafts of the PtDA produced by the research team. This expert panel assessed the PtDA based on: (1) member’s experience as patients who were making or who had made the decision about starting insulin treatment; (2) healthcare professionals who had helped patients in making such decisions; and (3) key opinion leaders/policy makers who were involved in implementing health services for diabetes in General Practice. Members of the review panel comprised two patients, three healthcare professionals, three key opinion leaders and three representatives from the local Primary Care Trust and research network.

We conducted the literature review for two reasons; firstly, we aimed to identify the effective decision support interventions tools that were available; secondly, we wanted to search for latest clinical evidence on the pros and cons of the different treatment options to be incorporated into the PtDA. Therefore, two systematic review were conducted. As far as possible, we searched for systematic reviews and national or local clinical practice guidelines. In the event that they were not available, findings from individual studies were appraised, synthesised and summarised. The quality of the information was graded according to the “grading for evidence-based rheumatology” guidelines [13].

A qualitative methodology using individual in-depth interviews and focus group discussion was used for the needs assessment, acceptability and feasibility studies. A mixed methodology was used in this process to triangulate the data so that the PtDA could cater for the comprehensive needs of the users. Patients with type 2 diabetes who were at the point of deciding whether or not to start insulin therapy were recruited into the study. Healthcare professionals, including general practitioners, practice nurses and dieticians, who were involved in managing patients with type 2 diabetes, were also included in the study. For the needs assessment, the users were interviewed individually for their needs when making (or facilitating) a decision on insulin therapy. As for the feedback on the acceptability and feasibility of using the PtDA, both the patients and clinicians were interviewed after they had used the PtDA at the consultation.

One of the researchers (CJN) conducted all the interviews using a semi-structured interview guides, which were developed separately for patients and clinicians based on the literature review and expert opinion (Additional files 1 and 2). The interviews were audio-recorded, transcribed verbatim, checked and analysed using a thematic approach. NVivo 7 software was used to manage the qualitative data.

In addition, a bespoke questionnaire was used to capture quantitative feedback from the users on the acceptability and feasibility of using the PtDA. The users provided general and specific feedback on the content, layout and usability of the PtDA using a five-point Likert scale.

## Results

### UKMRC and IPDAS frameworks

Table 3 demonstrated step-by-step how the findings from the needs assessment study, clinical evidence and the ODSF were incorporated into the development of the PtDA using the UKMRC framework. Both the UKMRC framework and IPDAS guideline guided the initial stages of the development of the PANDAs PtDA which included: convening an expert panel, comprising key stakeholders; conducting a needs assessment study; and searching for evidence for decision support interventions. The UKMRC framework highlights the importance of using theories to inform the development of complex interventions and is used to identify appropriate decision making theories. The drafting and piloting of the PtDA was also an important step in the UKMRC framework. The IPDAS framework guided the content and format development of the PtDA (Table 2)

**Table 3:** **The development of the PANDAs insulin PtDA based on needs, evidence and theory**

**Five steps in decision making**	**Description**	**Needs based**	**Evidence based**	**Theory based**
**1. Background information**	• This section aims to provide information about the decision of starting insulin; how diabetes could affect the patient personally; adherence to current diabetes treatment; and the range of treatment options available.	• In the needs assessment study, the patients wanted and the clinicians felt that the patients needed information on: insulin and its pros and cons; particularly the impact on their lives, symptoms, long term complications,	• The information on the benefits and risks of insulin was based on the NICE guidelines [14], UKPDS study [12]	• The ODSF proposed that patients should be ‘knowledgeable about the issues (of treatment)’ so that they would make an informed decision.
		• ‘Make no change’ was rarely offered to the patients; the healthcare professionals recognised that this was an option some patients preferred.		• A description of insulin therapy, pros and cons, its impact on quality of life, in sufficient detail during the first part of the PtDA would familiarise patients who were previously unfamiliar with the decision [15].
**2. Learn about the choices**	• This section describes each of the three treatment choices in detail, including the advantages and disadvantages of each option	• The selection of the information regarding the advantages and disadvantages of each treatment options was based the patients’ decisional needs and what the healthcare professionals felt was important for the patient to know before making the decision.	• The evidence used in this section (diabetic complications, hypoglycaemia, weight gain) were derived from best available evidence.	• Two theories were used when developing this section of the PtDA: the ‘theory of expected utility’ and the ‘prospect theory’.
	• The patients’ chance of getting diabetic complications is personalised according to their HbA1c.		• However, the strength of available evidence varied and this was reflected in the ‘evidence battery indicator’.	• The outcome probabilities presented in this PtDA include the risk of diabetic complications and the chance of experiencing the side effects of insulin.
	• The level of evidence is graded and presented as ‘number of bars in a battery’.	• The risk of complications of poor glycaemic control is personalised according to patients’ individual HbA1c.		• The risk of diabetic complications is presented both as the chance of the patient ‘getting the complications’ (‘negative framing’) as well as ‘not getting the complications’ (‘positive framing’).
**3. Thinking about what is important to you**	• This section clarifies patients’ values attached to the attributes of each treatment option.	• The patients had expressed the need for the healthcare professionals to address their concerns about insulin injections and the side effects.		• The ODSF proposes the importance of supporting patients in clarifying their values to ensure that the treatment option patients choose are in congruence with what is important to them (values).
	• They were asked to indicate whether the reasons for ‘choosing insulin’ and ‘not choosing insulin’ were important to them.	• This section on value clarification helped the healthcare professionals to understand patients’ values and priorities.		
		• The ‘reasons for choosing insulin’ reflected what the healthcare professionals felt was necessary for the patients to be aware of before making their decision.		• The ‘value clarification exercise’ in this PtDA focuses on the values related to the advantages and disadvantages of starting or not starting insulin.
**4. What else do you need to help you make a decision?**	• This section explores the support the patient needs before including information, values, support from the family and clinician, and certainty about the diagnosis	• The patients wanted the healthcare professional to address their concerns about the treatment options before making a decision.		• The ODSF postulates that a PtDA should address patients’ decisional needs which include: balanced and accurate information; clarity about values associated with the treatment options; support from healthcare professionals, family and friends to reduce their decisional conflict.
		• The healthcare professionals wanted to know why the patients were hesitant to start insulin.		
		• This section was designed to help bridge this gap by allowing the patients to communicate their concerns to the healthcare professionals effectively.		• This section of the PtDA was developed based on this theoretical framework.
**5. What’s next?**	• This is the final step and it asks the patients to indicate whether they are ready to make a decision and, if so, which option they preferred.			
	• For those who chose‘ add insulin’ as the option, they would complete an additional section which explored: their motivation; self-efficacy; barriers and facilitators in starting insulin.			

.

### Convening an expert panel

Initially, development and expert panels were convened to inform and advise on the development and evaluation of the PtDA. The development panel members were three healthcare professionals (2 GPs and a clinical nurse specialist in diabetes), one patient educator, three experts in decision science, two experts in evidence-based medicine and three experts in research methodology who had extensive experience in qualitative, quantitative and mixed methodologies. They were responsible for assessing the appropriateness of the research methodology used in the development and evaluation of the PtDA which informed the design and data analysis of the needs assessment acceptability and feasibility studies.

### Needs assessment

The needs assessment study identified four main needs common to both patients and clinicians: decisional, information, emotional and social support needs. For example, the patients wanted information on different treatment options available (besides insulin), the benefits and side effects of insulin therapy, and the impact of starting insulin on their lives. This information was incorporated into the PtDA. Although the PtDA did not address patients’ social and emotional needs directly, they were highlighted to the healthcare professionals who would address them during the consultation.

### Identify a theoretical framework

**Figure 2:**
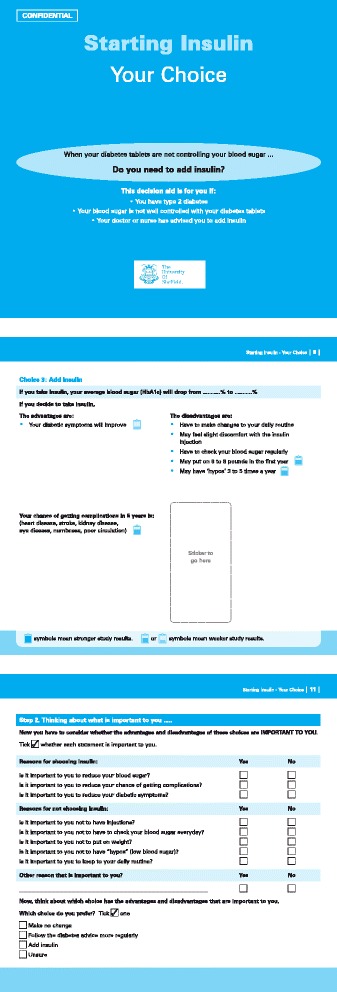
**An illustration of the PANDAs patient decision aid.**

Durand [11] identified a range of decision making theories that have been used to develop PtDAs, out of which, the Ottawa Decision Support Framework (ODSF) was the most commonly used and implemented. The ODSF is a conceptual framework which consists of a few theories such as expected utility theory, decisional conflict theory and social support theory [16]. The PANDAs PtDA was developed based on the ODSF, which postulates that by identifying an individual patient’s decisional needs, a PtDA can provide personalised decision support to patients, and help patients make an informed and value-based decision about their health which in turn leads to a better health outcome ie. improved decisional quality [17] (Figure 2). The ‘decisional needs’ highlighted in the ODSF, such as decisional conflict, values, knowledge, expectations and support, were incorporated into the PANDAs PtDA.

### Collate background information and clinical evidence

The background information and clinical evidence included in the PtDA was based on the needs of the patients and the healthcare professionals; availability and quality of the evidence; and the relevance to the local population. The evidence regarding the outcome probabilities for each of the treatment options was not easily available. A systematic review of the literature was, therefore, carried out to search, appraise and synthesise the information necessary to be incorporated into the PtDA.

We searched the literature systematically to determine the effectiveness of insulin-oral glucose-lowering drugs combination in reducing HbA1c, diabetic symptoms, diabetic complications and death compared to oral glucose-lowering drugs alone; its safety profile and _the_ level of reduction in HbA1c_._ We included: systematic review, randomised and non-randomised trials; controlled and uncontrolled trials; cohort study. The inclusion criteria for the participants were: people with type 2 diabetes; blood sugar was not well controlled despite taking maximum oral glucose-lowering drugs; and insulin-naive. Two electronic databases, Cochrane Trials Register and Medline, were searched for eligible studies from 1950 to 2006. In addition, the reference lists and bibliographies of all relevant papers were searched for additional studies using the ‘snowballing’ method. This was performed on key articles till ‘reference saturation’. Publications by key authors were also searched and they were contacted for unpublished data. Only English-language publications were included in this review.

For the electronic database search, the following terms and strategy were used: diabetes mellitus, type 2, insulin, thirst, weight loss, weight gain, polyuria, nocturia, fatigue, infection, vision disorders, haemoglobin A, glycosylated, blood glucose, diabetes complications, mortality, death, mortality, hypoglycaemia, weight gain, lipodystrophy

CJN independently assessed the titles and abstracts of the identified studies. Where a clear decision could not be made on the basis of the title or the abstract, the study was considered relevant. Full text of all relevant studies was retrieved. The method sections of the retrieved articles were examined to assess whether they satisfied the inclusion criteria; uncertainty was resolved by asking a co-researcher for independent assessment.

### Drafting of the PtDA

The initial drafts of the PtDA were developed by the research team based on the IPDAS guideline [5] and the ODSF [16]. It went through a number of iterations before it was ready for alpha testing (Table 2).

### Alpha testing

In line with the IPDAS recommendations, the PtDA draft was reviewed by the review panel before being revised by the research team. This phase in the development of the PtDA was iterative and repeated until no new comments emerged, ie it had reached “thematic saturation” (Table 2).

### Assessing readability

The IPDAS guideline recommends that PtDAs should be ‘written at a level that can be understood by the majority of patients in the target group’ and it sets the readability threshold at grade 8 (English year 9) using readability test such as SMOG or Fry [6]. The readability of this PtDA was measured using the Readability Calculations v. 7.0 software (Micro Power & Light Co. of Dallas, Texas 2005) and the results showed SMOG score at grade 7.

### Beta testing

During the development of the PtDA, the PtDA drafts underwent three cycles of review by the users (nine patients and 14 healthcare professionals in total) and the expert panel as well as revisions by the research team. All members of the expert panel felt that the PtDA was acceptable in terms of content, format and readability prior to its’ use in clinical practice.

In the initial feasibility study, the healthcare professionals also provided feedback on how the PtDA could be delivered in usual general practice. Their major concerns were time constraints and a lack of familiarity with the use of the PtDA, which were addressed by conducting a training workshop for the healthcare professionals before they use it with their patients. This feedback was discussed during the meetings with the review panel and a concerted effort was made to incorporate these feedback into the final PtDA and its delivery.

### Finalising the PTDA

The expert panel met four times during the review/revision process to discuss the feedback from the patients and the healthcare professionals based on the findings from the in-depth interview analysis and “acceptability” questionnaire surveys. The key findings were summarised and sent to the expert panel at least one week in advance. During the subsequent meetings, each member took turns to comment on each section of the PtDA as well as the findings from the in-depth interviews in order to achieve a consensus.

The PtDA was also revised in turn by the research team based on the analysis of the interviews and the consensus of the review panel meetings. This reviewing-revising iterative process was terminated when there were no further comments emerging from users’ interviews and review panel meetings.

The final draft was internally assessed using the IPDAS quality criteria checklist and proof read before sending for graphic design and printing. We found that the PANDAs PtDA satisfied all the criteria of the IPDAS checklist. This was the final step in the development of the PtDA before it was subjected to formal evaluation.

## Discussion

The development of the PANDAs PtDA demonstrates that it is feasible to use a combination of the IPDAS and the UKMRC frameworks to develop a PtDA based on users’ needs, evidence and theories (ODSF). The IPDAS recommendations provided evidence-based guidance on the development of the content and structure of the PtDA; the users’ healthcare decisional, social and emotional needs informed the selection of the content; and the ODSF provided a theoretical basis to link the development of the PtDA to the outcome measures for evaluation. Finally, the UKMRC framework for developing and evaluating complex interventions in Primary Care provided an overarching framework to guide the iterative process, inform the choice of research methods and ensure that the PtDA could be successfully developed, evaluated and implemented in practice.

The recent IPDAS collaboration evidence document reviewed the PtDAs assessed in the latest Cochrane Review [4] and found that only half of the PtDAs underwent field testing with patients and clinicians in the ‘real world’ and only 17% documented the method of reviewing and synthesising clinical evidence [18]. It also highlighted the fact that few PtDAs provide sufficient details to assess whether or not they comply with the IPDAS criteria.

This study describes systematically how the IPDAS criteria guided the development of the PANDAs PtDA. The use of a theoretical framework to develop PtDAs was not part of the IPDAS standards [6]. However in the recent update on the IPDAS evidence document, the use of a theoretical framework to develop PtDAs has been proposed [19]. Similarly, the UKMRC guidance on how to develop complex interventions such as PtDAs also recommends the use of theories, which help to describe, explain and predict how the PtDA might work. With the growing number of clinical trials on PtDAs, a comparison between PtDAs which have or do not have a theoretical framework, is possible and the revised IPDAS standards on the evaluation of PtDA quality can be used to address this task [20].

The decision on ‘what’ and ‘how much’ to include in a PtDA is also a challenging one. Conventionally, PtDAs have focussed on delivering accurate and balanced information about the treatment options to patients ie emphasising the information needs [21]. However, increasingly, there is growing evidence that people do not make decisions based only on the information they receive [22]. Other considerations such as the availability of healthcare, emotional and social support may also affect how patients make decisions and it is, therefore, crucial that these needs of patients are attended to during the decision making process. In this study, the findings from the needs assessment reinforces this.

When identifying the best evidence to be included in the PtDA, we encountered a few difficulties. Firstly, there was a lack of systematic reviews or trials comparing insulin with ‘make no change’ or ‘lifestyle modification’. This is because it would be unethical and unacceptable to compare insulin therapy with no treatment in people with uncontrolled diabetes. There is also a lack of trials comparing insulin with lifestyle changes as most of these trials are funded by the pharmaceutical industry which have a vested interest in promoting drug usage [23].

In addition, there are also substantial variations in the reported outcomes of studies. For example, some studies report hypoglycaemic episodes as the number of episodes per 100 person year, whilst others report outcomes as “proportions of people experiencing hypoglycaemic episodes over a year”. In addition, few clinical trials report on outcomes that are important to patients, such as ‘symptom relief’, which are relevant to patients when making any sort of medical decision [24].

In the PANDAs PtDA a list of diabetic symptoms was included and patients were asked to indicate whether or not they had experienced any of the symptoms. Although the evidence was, to some extent, lacking, “diabetic symptom relief” was included the in the PtDA as this is advice that is routinely given by healthcare professionals and it has a plausible scientific basis. Future clinical trials should routinely report outcomes that are clinically important to patients.

In the PANDAs PtDA, the risks and benefits were communicated to patients using the accepted principles of risk communication such as using textual, number and visual display to present risks; using natural frequencies rather than percentages; and emphasising the interaction between patients and healthcare professionals [25]. There is an increasing recognition that people use both analytic and intuitive mechanisms to make decisions; so how people perceive risk may depend as much on logic and systematic analysis as well as emotions and heuristics [26].

Concerns were also raised about the purpose of the PtDA and whether it would be used to “convince” patients to start insulin treatment. Thompson PB has raised the same issue that risk communication might be used to encourage changes in peoples’ behaviour for the sake of public health gain [27]. However, the aim of the PANDAs PtDA was to help patients make a decision which was informed and based on what was important to them and this was achieved by ensuring that the risk communication tool was balanced, accurate and up to date.

The purpose of value clarification in the process of shared decision making is to help patients recognise that their values play an important role in decision making. It also helps patients to determine which treatment option and its attributes are important to them and share their values with the healthcare professionals and their families [28].

In the PANDAs study, an explicit “balanced technique” was used to help patients compare and indicate the relative importance of the treatment option as well as the attributes of each option [17]. However, patients found it difficult to answer the “negatively phrased” questions (e.g. ‘how important is it to avoid weight gain?’), especially when the response was also negative (eg ‘not important’). This difficulty was compounded when they were required to rate the importance (from ‘not important’ to ‘very important’). As a result, the questions asked in the value clarification section of the PtDA were modified and the response items were reduced to a yes/no option. It was clear that the accuracy of response to the questions in the PANDAs PtDA needed to be weighed against the ease of answering the questions.

The PtDA was delivered in a booklet based on feedback from the patients, healthcare professionals and expert panel during the needs assessment study. PtDAs can be developed in other formats including web-based compact discs or digital versatile disc; audio book, common decision board and patient focus groups. Increasingly web-based decision aids are preferred by developers. A paper-based PtDA was chosen because both healthcare professionals and patients during the feasibility study found the paper-based PtDA acceptable and the PtDA itself needed to be used by healthcare professionals and patients together in a consultation. In addition, a paper PtDA was portable and could be taken home as required by the patient for further consideration and it does not require any equipment. Most of the target population for the use of the PtDA were above 50 years of age and the assumption which was made for the study was that the majority of people in this age group prefer to use paper-based tools and are generally less familiar with using computers or the internet. However, this preference is changing [29]. A survey conducted among orthopaedic surgeons in the UK found that more than half of the respondents preferred a booklet as a PtDA to other media such as web-based PtDA, CD/DVD and audiotape [30].

However, the disadvantages of using a paper-based PtDA are that the latter requires printing and mailing which may hinder wide dissemination and printing and reprinting of the PtDA was costly. By comparison, developing a web-based PtDA often needs only a single payment for computer programming and web design in the initial phase and maintenance and updating of a web-based PtDA requires less cost than reprinting of the paper-based PtDA.

Finally, web-based or computer-assisted PtDAs allow data to be collected and, if needed, summarized - so that it can be fed back to the patients and healthcare professionals, an advantage which paper-based PtDAs do not have. Nevertheless, a patient decision aid, regardless of the mode of delivery, only serves as a tool to facilitate discussion on the decision; it is not meant to replace a clinical consultation.

### Strengths and limitations

Although the PANDAs PtDA was developed according to the two frameworks and, as far as possible, based on needs, evidence and theories, there were numerous challenges which we encountered during its development. There was some overlap in the roles of the members within the expert panels which had the potential of creating biases in the development process. However, both panels acknowledge the potential biases. On the other hand, it was found that there were strengths in dual roles which, for example, helped to facilitate communication among members of the panel who were from very diverse backgrounds.

Feldman-Stewart et al. (2007) [31] reported that patients are often not consulted about the information they would like to be included in the PtDA [31]. The strength of this study is that it involved users (both patients and healthcare providers) throughout the development of the PtDA using individual in-depth interviews, questionnaire surveys and an expert consensus to assess users’ needs; to draft and revise the PtDA; and to determine its acceptability and feasibility. The mixed methods approach was used to help ‘triangulate’ the data and improve the credibility of the development process [32].

## Conclusions

This study has demonstrated the feasibility of the IPDAS and the UKMRC frameworks for the development and evaluation of a PtDA. The IPDAS guideline was useful for informing the content, development and formatting of the PtDA, while the UKMRC framework helped to link the process of development with the evaluation and implementation of a complex intervention. Both frameworks are complementary and essential components to guide the development and evaluation of future PtDAs.

## Additional files

Additional file 1:
**Interview guide – patients.**
Additional file 2:
**Interview guide – healthcare professionals.**
