# Supplementary material for: A ‘combined framework’ approach to developing a patient decision aid: the PANDAs model
Source: BMC Health Serv Res. 2014 Oct 24;14:503. doi: 10.1186/s12913-014-0503-7 (PMC4210601; doi:10.1186/s12913-014-0503-7)
Supplement: Additional file 1: — Interview guide – patients. [file 12913_2014_503_MOESM1_ESM.doc]

**Personal Interview Questions for Key Informants**

**– CONSUMER/PATIENT VERSION**

(modified from Ottawa Decision Support Needs Assessment tool)

**INTRODUCTION**

Good morning/afternoon/evening. My name is ___________________of University of Sheffield and I am involved with Professor Nigel Mathers in conducting a survey to learn more about the needs of people when they are making decisions about the treatment of diabetes.

This information will help us to develop better educational materials for people facing these decisions.

All of the information we collect in this survey will be kept confidential. We’d like your help and it won’t take more than 15 minutes.

**DECISION**

1. **At this time, what do you think are the most important decisions patients with type 2 diabetes face?**
2. **Let’s focus on one particular decision: whether to start insulin.**

| 1. **Let’s talk about the difficulty people have making this decision about starting insulin. How do you feel when making this decision?**   **_______________________________________**  **_______________________________________**  **_______________________________________**  **_______________________________________**  **_______________________________________**  **_______________________________________**  **_______________________________________**  **_______________________________________**  **_______________________________________** | ***Probe behavioural manifestations of decision conflict***  **Do you feel:**   unsure about what to do?   worried what could go wrong?   distressed or upset?   constantly thinking about the decision?   wavering between choices or changing  their mind?   delaying the decision?   questioning what is important to them?   feeling physically stressed, tense  muscles, racing heartbeat, difficulty  sleeping? |
| --- | --- |
| 1. **What makes this decision difficult for you?**   **_______________________________________**  _______________________________________  _______________________________________  _______________________________________  _______________________________________  _______________________________________  _______________________________________  _______________________________________  _______________________________________  _______________________________________  _______________________________________  _______________________________________  _______________________________________ | *Probe factors contributing to decisional conflict*  **Are you:**   lacking information about options,  benefits and risks?   lacking information on the chances of  Benefits and harms?   confused from information overload   unclear about what is important to them?   feeling unsupported in decision making?   feeling pressure from others?   lacking motivation or not feeling ready to  make a decision?   lacking the ability or skill to make a  decision? |

1. **Thinking about this decision, what are the options that you have?**
2. **What do you see as the main advantages/benefits and disadvantages/risks of the options?**

| **Option** | **Advantages/Benefits** | **Disadvantaged/Risks** |
| --- | --- | --- |
| 1. |  |  |
|  |  |
|  |  |
|  |  |
| 2. |  |  |
|  |  |
|  |  |
|  |  |
| 3. |  |  |
|  |  |
|  |  |
|  |  |

| 1. **What role do you prefer to play when making this decision?**   ___________________________________  **___________________________________**  **___________________________________**  **___________________________________**  **___________________________________**  **___________________________________**  **___________________________________**  **___________________________________**  **___________________________________** | ***Probe role:***  **Do you prefer:**   the practitioner to make the decision for  you?   to share the decision with the practitioner?   to make the decision on your own? |
| --- | --- |

| 1. **Who else besides your practitioners is involved in making this decision?**   ________________________________________  ________________________________________  ________________________________________  ________________________________________  ________________________________________  ________________________________________  ________________________________________ | ***Probe:***   spouse   family   friend   health care provider   others, specify: _______________  ______________________________ |
| --- | --- |

| 1. **What is their role in making this decision (i.e. the person mentioned above)?**   ________________________________________  ________________________________________  ________________________________________  ________________________________________  ________________________________________  ________________________________________  ________________________________________ | ***Probe:***  **Do they usually:**   make the decision for you?   share the decision with you?   provide support or advice to  you but let you make the decision on  your own?   Don’t know   Others, specify: _______________  ______________________________ |
| --- | --- |

| 1. **How do you go about making this decision?**   ________________________________________  ________________________________________  ________________________________________  ________________________________________  ________________________________________  ________________________________________  ________________________________________ | ***Probe decision making behaviour:***  **Do you:**   get information on options?   get information on the chances of benefits and risks?   consider the personal importance  of the benefits and risks?   get information on how others go  about deciding?   get support from others?   find ways to handle pressure? |
| --- | --- |

1. **What would help you to make this decision?**

________________________________________________________________________________________________________________________________________________________________________________________________________________________________________________________________________________________________________________________________________________________________________

1. **What will get in the way when you are making this decision?**

________________________________________________________________________________________________________________________________________________________________________________________________________________________________________________________________________________________________________________________________________________________________________

1. **Is there anything else that would help you to overcome the barriers to decision making?**

________________________________________________________________________________________________________________________________________________________________________________________________________________________________________________________________________________________________________________________________________________________________________

1. **I will list some ways to help people with making a decision. Which ones do you think might be useful to you?**

| **Options** | **Content** |
| --- | --- |
|  **Counselling from a**  **health practitioner** | If yes, specify what type of health practitioners. |
|  **Discussion groups of**  **people facing the same**  **decision** | If yes, specify what type of organization or group |
|  **Information materials** | If yes, specify content:   Health condition   Options   Benefits   Risks   Probabilities of benefits/risks   Help considering the personal importance of benefits  versus risks   Guidance in the steps of deliberation and communication   Others, specify: ________________________________________________ |
|  | If yes, specify format:   Booklet, pamphlets   Internet   Videos/DVDs   Other, specify: _________________________________________________ |
|  | If yes, who do you think should prepare information about the decision:   Pharmacies   Expert medical and health practitioners   Health societies for specific condition (e.g. Diabetes UK)   Government   Companies that produce and sell drugs and health products   Consumer associations   For profit companies that produce health information  (e.g. BMJ Best Treatment) |

1. **Is there anything else that would help you to make a decision?**

________________________________________________________________________________________________________________________________________________________________________________________________________________________________________________________________________________________________

**PATIENT’S PARTICULARS**

1. **Age**

 Twenties

 Thirties

 Forties

 Fifties

 Sixties and above

1. **Gender**

 Male

 Female

1. **Ethnicity: _________________________________________**
2. **Education: _____________________________________________________**
3. **Occupation: ____________________________________________________**
4. **Duration of Diabetes: __________ years**
