# Supplementary material for: A ‘combined framework’ approach to developing a patient decision aid: the PANDAs model
Source: BMC Health Serv Res. 2014 Oct 24;14:503. doi: 10.1186/s12913-014-0503-7 (PMC4210601; doi:10.1186/s12913-014-0503-7)
Supplement: Additional file 2: — Interview guide – healthcare professionals. [file 12913_2014_503_MOESM2_ESM.doc]

**Insulin Decision Aid Interview Guide – Healthcare Professionals**

*Part 1 – Needs Assessment for Decision Making*

**General – Starting Insulin**

1. Can you please tell me the profile of type 2 diabetic patients that you manage in your practice?
2. What decisions do these patients have to make?

*Let’s focus on: whether to start insulin.*

**Patient’s Decision Making**

1. Is starting insulin a difficult decision for your patients?
2. Why is it difficult for them? What difficulties do patients face when deciding whether to start insulin?
3. How do they feel when they are making this decision?
4. What are the things that patients consider before they decide whether or not to start insulin? (information, what information, values, how others do it, support from others, handling pressure)
5. What kind of help do they need to make this decision? (information, support)
6. Who else, besides your patient, are involved in making this decision? (family and friends; their role)

**GP’s Support**

1. Who are the patients whom you would advise to start insulin?
2. If a patient refuses to start insulin, what other options do they have? What are the advantages and disadvantages of these options?
3. How do you help patients to start insulin?
4. What role do you play in the decision making process?
5. How do you explain the risks and benefits of insulin to your patients?
6. What barriers do you face when advising them to start insulin? (talking about risks and benefits, exploring values, ICE, support, coaching them how to make decisions, motivating them,
7. What kind of help do you need to overcome these barriers?

**Decision Support**

1. Here are some ways to help patients make decisions – what do you think?
   - Counselling from healthcare professionals
   - Discussion group of people facing the same problem
   - Information materials – content, format and who should prepare these information

*Part 2: Feedback on Patient Decision Aid*

1. General opinions about the decision aid
2. Based on their response to the questionnaires, discuss their specific opinions about the decision aid: amount of information, balance, clarity, being helpful and whether they would recommend to others.
3. Who are the patients whom you think would benefit from this decision aid?
4. When would you use this decision aid in your practice?
5. How would you use it?

**End of Interview**
